# Clinical, biochemical and molecular characteristics of classic homocystinuria in Saudi Arabia and the impact of newborn screening on prevention of the complications: A tertiary center experience

**DOI:** 10.1002/jmd2.12454

**Published:** 2024-11-11

**Authors:** Ahmed Sarar Mohamed, Talal AlAnzi, Amal Alhashem, Hadeel Alrukban, Fahad Al Harbi, Sarar Mohamed

**Affiliations:** ^1^ College of Medicine The National University Khartoum Sudan; ^2^ University Hospitals Birmingham NHS Foundation Trust Birmingham Heartlands Hospital Birmingham UK; ^3^ Department of Pediatrics Prince Sultan Military Medical City (PSMMC) Riyadh Saudi Arabia; ^4^ Newborn Screening Laboratory Prince Sultan Military Medical City (PSMMC) Riyadh Saudi Arabia; ^5^ Department of Pediatrics, College of Medicine Al Faisal University Riyadh Saudi Arabia; ^6^ Prince Abdullah Bin Khaled Coeliac Disease Research Chair College of Medicine, King Saud University Riyadh Saudi Arabia

**Keywords:** amino acid disorder, classic homocystinuria, inborn errors of metabolism, newborn screening, sulfur

## Abstract

**Background:**

Classic homocystinuria (HCU) is a rare inborn metabolic disease that is generally asymptomatic at birth. If untreated, it can cause a wide range of complications including intellectual disability, lens dislocation, and thromboembolism. This study aimed to describe the natural history and the molecular findings of patients with HCU, and to assess the importance of early diagnosis.

**Methods:**

This study retrospectively collected data on patients attending the metabolic unit at Prince Sultan Military Medical City, Riyadh, Saudi Arabia from 2011 to 2024. Demographic, clinical, and molecular data was extracted from the electronic medical records.

**Results:**

Among the 33 patients with HCU enrolled, 5/33 (15%) were diagnosed by newborn screening and the rest were diagnosed on clinical grounds. The complication profile was vast, with neuropsychiatric, musculoskeletal, ophthalmic, and thromboembolic morbidities complicating the disease course in 28/33 (85%) of the patients. None of the newborn screened patients had complications while all of the non‐newborn screened patients had at least one complication, *p* < 0.0001. The majority of parents in this cohort were highly consanguineous, with 90% had first or second cousin marriage. Seven previously reported variants were detected in this cohort and one novel variant was found in three patients (c.828+2‐828+ 3 delins ACACTTGCATCC, p.?). The known pathogenic variant (c.969G>A, p. (Trp323*)) was seen in most of the patients, with all of them coming from one tribe.

**Conclusions:**

This cohort gives further evidence that the newborn screening for HCU is likely to prevent the complications associated with the disease at least in the first few years of life. Therefore, newborn screening for HCU should be encouraged. Our molecular studies revealed the presence of a founder variant, detected in patients from a single tribe. This suggests that specific mutation testing may be cost‐effective for individuals from certain ethnicities.

**Synopsis**

In this cohort, patients with classic homocystinuria who were diagnosed by newborn screening exhibit none of the complications associated with this disorder while all of the patients with classic homocystinuria who were diagnosed on clinical grounds developed at least one of the complications including ophthalmic, neuropsychiatric, skeletal and thromboembolism.

This article gives further evidence that newborn screening for HCU is likely to prevent the complications associated with this disease.

## 1 INTRODUCTION

Classic homocystinuria (HCU) is the most common disorder of sulfur amino acid metabolism.^1^ It is an autosomal recessive disease caused by biallelic pathogenic variations in *CBS* gene which encodes for the enzyme cystathionine β‐synthase (CBS).^1^

Methionine, a sulfur containing amino acid, is metabolized to homocysteine (Hcy) by transmethylation facilitated by the expression of methionine adenosyltransferase (MAT) I/III and glycine *N*‐methyltransferase (GNMT) in the liver.^2^ The further metabolism of Hcy by the transsulfuration pathway is facilitated by activation of CBS by *S*‐adenosylmethionine (SAM).^2^ The Hcy produced by transmethylation can either undergo remethylating, which converts Hcy back to Methionine, or transsulfuration, which is responsible for Hcy catabolism and Cysteine synthesis.^2^

The CBS enzyme uses pyridoxal 5′‐phosphate as a cofactor.^2^ Therefore, CBS deficiency is classified into two phenotypes depending on vitamin B6 responsiveness. Pyridoxine responsiveness is thought to depend on the extent to which the CBS mutations affect the cofactor binding site.^3^

Worldwide prevalence of HCU based on diagnosis of symptomatic individuals is 0.82:1 00 000, while that based on neonatal screening by MS/MS was 1.01:1 00 000 newborns.^4^

In the Gulf region and in Qatar particularly the reported incidence of classic pyridoxine unresponsive HCU is (1:3000) in the Qatari population.^5^ An incidence of 1:50 000 and of 1:43 000 was reported among Kuwaiti newborns^6^ and a similar incidence was reported in the eastern region of Saudi Arabia.^7^

Patients with CBS deficiency vary markedly in their symptoms, age of onset and rate of progression of clinical signs. Generally, pyridoxine‐responsive patients have a milder phenotype and a later onset than the pyridoxine unresponsive ones.^3^ The major clinical manifestations of CBS deficiency include dislocation of the optic lenses and/or severe myopia.^8^ Other manifestations include excessive height and length of the limbs (‘marfanoid’ habitus), osteoporosis and bone deformities, such as pectus excavatum or carinatum, genu valgum and scoliosis.^3^ HCU may also be complicated by developmental delay/intellectual disability, seizures, psychiatric and behavioral problems and extrapyramidal signs.^8^

According to the European guidelines,^8^ plasma total Hcy constitutes the frontline test for HCU, and levels above 100 μmol/L, together with elevated methionine are highly suggestive of the diagnosis which needs to be established by molecular testing.^8^

Current treatment options aims at lowering the plasma Hcy by methionine restricted diet, betaine, folic acid and pyridoxine supplementation.^3^ Betaine (*N*,*N*,*N*‐trimethylglycine) acts as a methyl‐donor for homocysteine to form methionine by the enzyme betaine‐homocysteine S‐methyltransferase.^3^, ^8^ Therefore, betaine lowers Hcy level and improve the metabolic control of patients with HCU. The dietary management of HCU is based on protein/methionine restriction that decreases the influx in homocysteine metabolism and thereby lowers its level.^9^

Recently, there are different treatment modalities and an ongoing research studies concentrating on new and novel treatment options for HCU like enzyme replacement therapy, chaperones, probiotics, liver transplant and gene therapy.^9^, ^10^, ^11^ An emerging preclinical data demonstrated the efficacy of an orally administered enzyme therapy that could degrade methionine within the small intestine and therefore decreases the plasma serum Hcy level.^11^

Universal newborn screening (NBS) for HCU, whereby methionine levels are measured by tandem mass spectrometry, aiming at early detection.^5^, ^6^, ^7^, ^8^ However, false negatives with the current NBS procedures are challenging.^5^, ^6^, ^7^, ^8^ Suggested methods to increase the NBS sensitivity include reduction of methionine cut‐off, the use of Hcy as a secondary tier, and introduction of molecular screening.^5^, ^6^, ^7^, ^8^ Inclusion of HCU in the NBS programs has substantially contributed to the improvement in the long‐term outcomes.^12^ The early start of treatment to maintain low levels of Hcy seems to prevent the complications of HCU.^10^ We were the first center in Saudi Arabia to include HCU in newborn screen aiming at reducing long term disabilities.^13^

In this paper, we retrospectively reviewed the characteristics and the clinical course of all patients with HCU evaluated in the division of genetics and metabolic medicine at Prince Sultan Military Medical City in Riyadh, Saudi Arabia. We compare the outcome in patients detected by NBS to those diagnosed on clinical grounds.

## 2 MATERIALS AND METHODS

This is a retrospective study conducted at the tertiary metabolic center at Prince Sultan Military Medical City (PSMMC), Riyadh, Saudi Arabia. It is the metabolic referral center for over 20 military hospitals in the country. The center receives patients' referrals with suspected metabolic and genetic conditions from all military health facilities and it houses the main biochemical laboratory for the national newborn screening program (NBS) for all military hospitals in the Kingdom. The Team processes, interprets and manages all NBS samples for babies born in all military hospitals in Saudi Arabia.

We collected the data of all HCU patient's seen in our center from 2011 until 2024. The data was extracted from the electronic medical records and includes demographic, clinical, biochemical and molecular characteristics using data collection form. Pertinent clinical findings presenting features and complications were collected.

### 2.1 Data collection and diagnostic workup

The patients recruited in this study were classified into two groups. A group diagnosed by NBS and the second group diagnosed by non‐NBS, based on clinical presentation or positive family history. The latter group includes all patients diagnosed on clinical grounds such as presentation with symptoms and signs suggestive of HCU or as a result of family screening of siblings after an index patient was diagnosed.

Patients who have had high methionine and total homocysteine levels, either due to newborn screening or as a result of clinical suspicion of HCU were confirmed by genetic testing. This included targeted mutation studies (when familial variant is known), CBS single gene sequencing, or whole exome sequencing (WES).

#### 2.1.1 Clinical, biochemical and treatment data

The collected clinical data covered the details of the four main complications categories, namely thromboembolic, neuropsychiatric, musculoskeletal and lens dislocation/subluxation and refractive errors. Neuropsychiatric complications mainly comprise intellectual disability and attention deficit hyperactivity disorder (ADHD). Musculoskeletal disorders include osteoporosis and marfanoid features. Thromboembolic complications include deep vein thrombosis and cerebral venous thrombosis. Radiological findings in brain MRI, echocardiography, carotid doppler and DEXA scan were also collected. Furthermore, we reviewed and gathered the serial biochemical measurements of total homocysteine and methionine level as marker of the disease control along with the treatment details. The treatment medications are betaine, pyridoxine, folic acid and hydroxocobalamin in addition to the dietary intervention of low‐methionine special formula.

#### 2.1.2 Molecular study data

The collected molecular data included the targeted mutation study, CBS single gene sequencing, or WES. These tests were performed as previously described.^14^ The specific genetic test was requested by the treating physician according to the family history, the clinical features and the availability of the tests at the time of presentation. These test were performed in a clinical diagnostic laboratory where DNA was extracted from blood collected in EDTA tube, sequencing was performed^14^ and variants were classified as described in the ACMG guidelines.^15^ The testing methodology varied based on the selected analysis, CBS gene familial variant analysis, carrier testing, was performed via sequencing both DNA strands of the relevant CBS targeted region.^14^ CBS gene testing was conducted through Sanger sequencing and CNV analysis.^14^ Whole exome sequencing was performed via targeting >98% of the coding RefSeq from the human genome build GRCh37/hg, as well as the mitochondrial genome.^14^ The generated library is sequenced to obtain at least 20× coverage depth for >98% of the targeted bases.^14^, ^15^ These different testing methods were used based on the availability of the resources and cost‐effectiveness and new case versus previous family member affected.

### 2.2 Statistical analysis

Data on specific socioeconomic, clinical, biochemical and molecular variables were tabulated. The Statistical Package for Social Sciences, version 22.0 (IBM Corp, Armonk, NY, USA) was used for analysis. Categorical variables were shown as percentages and frequencies, while continuous variables were presented as mean and standard deviation. Chi‐squared test was used to assess for statistical significance in categorical variables, whereas Welch's unpaired *t*‐test was used to assess for statistical significance for the continuous variables in Table 2, and the unpaired *t*‐test (two‐tailed) was used for the continuous variables in Table 5. A *p‐*value <0.05 was considered significant.

## 3 RESULTS

### 3.1 Patient characteristics

This retrospective descriptive study included data collected from 33 patients with genetically confirmed classic HCU, 16 of whom were male and 17 were female (Table 1).

**TABLE 1:** Clinical characteristics of 33 patients with classic homocystinuria.

Variable	Number
Male (%)	16/33 (48%)
Female (%)	17/33 (52%)
Consanguinity (%)	30/33 (91%)
Newborn screening diagnosis (%)	5/33 (15%)
Non‐newborn screening diagnosis (%)	28/33 (85%)
Number with uncomplicated course (%)	5/33 (15%)
Number with complicated course (%)	28/33 (85%)

Five patients (15%) were diagnosed by NBS while 28/33 patients (85%) were diagnosed by non‐NBS method which is based on clinical ground such as presentation with symptoms and signs suggestive of HCU, complications suggestive of HCU and/or positive family screening of siblings after an index patient was diagnosed (Table 1). The rate of consanguinity in the study group was high (91%). Compared to patients diagnosed on NBS, those who were not diagnosed by NBS tend to be older at the time of the diagnosis (*p* < 0.0001) and had longer duration of follow up (*p* < 0.002), Table 2.

**TABLE 2:** Duration of follow up among newborn screened and non‐newborn screened patients with homocystinuria.

Method of diagnosis	Age at last follow‐up (mean ± SD)	Age at diagnosis (mean ± SD)	Duration of follow‐up (mean ± SD)
Newborn screening	3.6 ± 1.0 years	0.0 ± 0.0 years	3.6 ± 1.0 years
Non‐newborn‐screening	15.4 + 4.7 years	8.9 ± 4.1 years	6.6 ± 4.0 years
*p* value	<0.0001	<0.0001	0.0020

### 3.2 Complications

Of the 33 patients with HCU enrolled, 28 had complications (85%). All the 28 patients who were not diagnosed by NBS had at least one or more complications while none of those diagnosed on NBS had any complication, *p* < 0.0001 (Table 3).

**TABLE 3:** Outcome among newborn screened and non‐newborn screened patients with homocystinuria.

Method of diagnosis	Number of patients with complications
Newborn screening	0/5 (0%)
Non‐newborn‐screening	28/28 (100%)
*p* value	<0.0001

Lens dislocation/subluxation was the most common complication observed in 23/33 patients. It was documented on the initial presentation in 13/33.

The neuropsychiatric complications seen in 16/33 patients and documented on half of them on first presentation.

The musculoskeletal complications were observed at the initial presentation in 11/33 patients.

**FIGURE 1:**
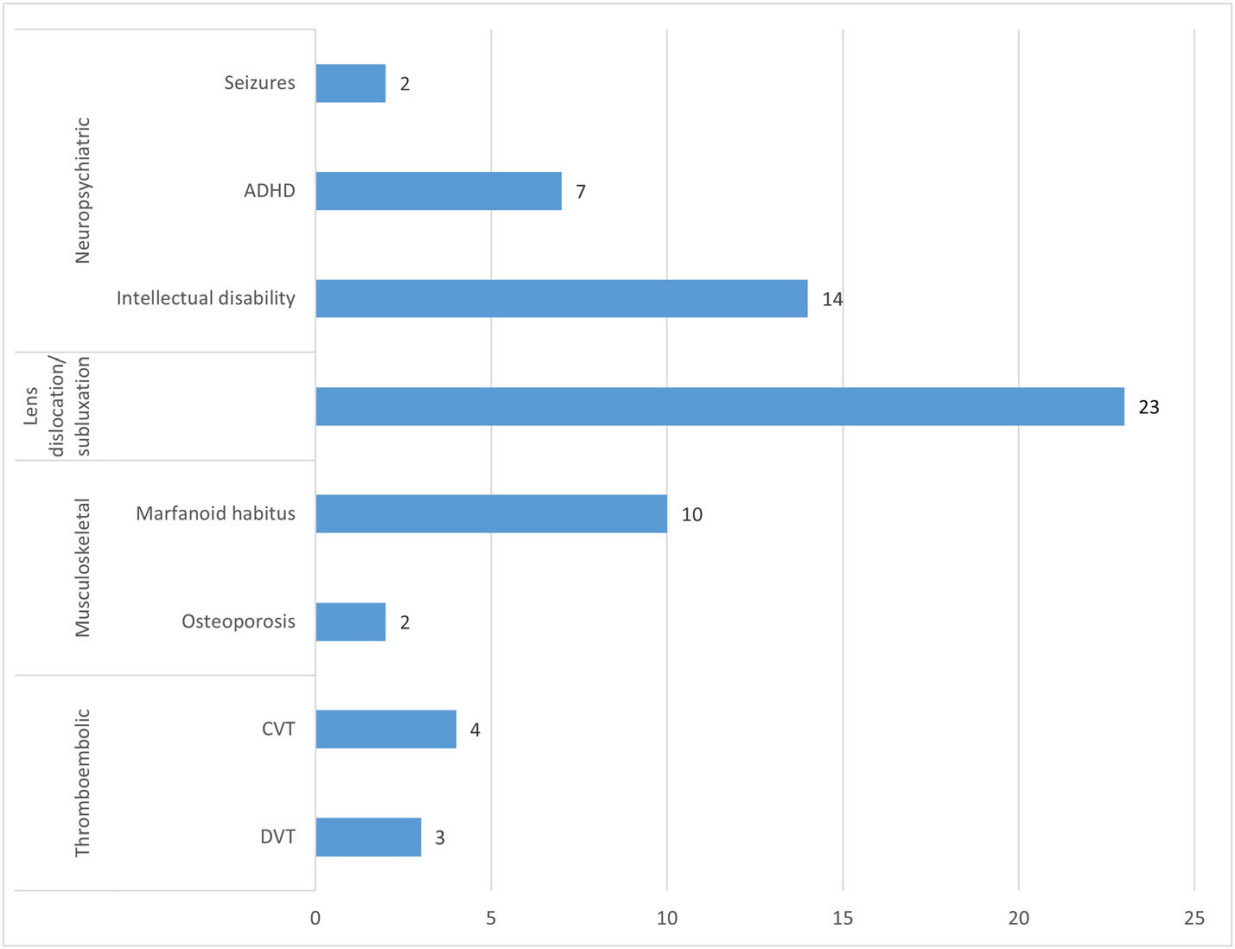
Complications profile of 28 patients with classic homocystinuria diagnosed by non‐newborn screening method. CVT, cerebral venous thrombosis; DVT, deep venous thrombosis.

Thromboembolism complicated the course of 7/33 patients and was the initial presentation in two of them that led to the suspicion and the final diagnosis of HCU. The complication profile is shown in Figure 1 and Table 4.

**TABLE 4:** Pattern of complications among newborn screened and non‐newborn screened patients with homocystinuria.

Diagnosis	Neuropsychiatric complications	Musculoskeletal complications	Lens dislocation/subluxation	Thromboembolic complications
NBS diagnosis (5)	0/5 (0%)	0/5 (0%)	0/5 (0%)	0/5 (0%)
Non‐NBS diagnosis (28)	16/28 (57%)	11/28 (39%)	23/28 (82%)	7/28 (25%)
*p* value	0.0093	0.043	0.0001	0.10

The age onset of HCU complications was variable with one patient who was diagnosed at 11 years of age having all the four aforementioned categories of complications, including learning difficulties, CVT, lens dislocation and musculoskeletal manifestations, and another patient, who was diagnosed 9 years ago, having no complication.

#### 3.2.1 Ophthalmic complications

Of the 23/33 patients (70%), 21 had lens dislocation and 2 had lens subluxation. As expected, refractory visual defects constituted a significant source of morbidity, occurring in 11/33 patients (33%), all had lens dislocation or subluxation.

#### 3.2.2 Neuropsychiatric complications

The neuropsychiatric complications occurred in 16/33 patients and documented in 8 patents at their initial presentation. The main neuropsychiatric complication was intellectual disability followed by ADHD (Figure 1). Some of the patients had both intellectual disability and ADHD. Developmental and intellectual assessment was performed clinically by the treating physicians for all patients. Only four patients had formal intellectual quotient (IQ) assessment performed by trained psychologist using appropriate scales. Three of these patients had IQ less than 70 and the fourth one 84. The findings of these formal IQ evaluations were consistent with the treating physician clinical judgments.

#### 3.2.3 Thromboembolic complications

Another cause for concern in the patients was thromboembolic complications, occurring in 7/33 (21%) patients, with 4 (12%) suffering from cerebral venous thrombosis and 3 (9%) having deep vein thrombosis, one of which necessitated an above‐knee amputation (Figure 1).

#### 3.2.4 Musculoskeletal complications

The musculoskeletal abnormalities among our patients comprised marfanoid features and osteoporosis, which together complicated the clinical course of 11/33 patients (33%), with one patient having marfanoid change together with osteoporosis, taking the total number of musculoskeletal complications to 12 as shown in Figure 1. Marfanoid complications occurred in 10/33 patients (30%). Of these patients, three patients had spinal deformities, which included kyphotic, scoliotic and lordotic deformities. Osteoporosis was featured in 2/33 patients (6%), and a further two manifested osteopenia on bone scan.

### 3.3 Diagnostic studies

#### 3.3.1 Total homocysteine levels

Methionine and total Hcy levels were serially measured and recorded for all patients, and the mean levels elicited over the follow‐up period ranged widely, from 14.5 to 864.3 μmol/L for methionine and from 45.4 to 748.3 μmol/L for total Hcy. As shown in Table 5, patients who were diagnosed by newborn screening had better biochemical control as shown by the mean total Hcy levels during follow up, this was statistically significant. The initial and the last total Hcy and methionine levels were not statistically different between the two groups (Table 5).

**TABLE 5:** Biochemical profile of 33 patients with homocystinuria according to the method of diagnosis.

Diagnosis	Mean initial methionine level ± SD, μmol/L	Mean last methionine level ± SD, μmol/L	Mean methionine level during follow‐up ± SD, μmol/L	Mean initial total homocysteine level ± SD, μmol/L	Mean last total homocysteine level ± SD, μmol/L	Mean total homocysteine level during follow up ± SD, μmol/L
All patients	401 ± 254	588 ± 281	499 ± 210	303 ± 266	182 ± 133	219 ± 137
NBS diagnosis (5)	270 ± 209	547 ± 564	333 ± 326	156 ± 151	136 ± 85	107 ± 45
Non‐NBS diagnosis (28)	426 ± 258	596 ± 217	531 ± 174	330 ± 275	191 ± 139	240 ± 139
*p* value	0.26	0.75	0.075	0.18	0.40	0.045

#### 3.3.2 Molecular studies

Genetic tests comprised whole exome sequencing, done in 19 patients, and CBS gene sequencing, which was done in 12 patients, as well as carrier testing which was the means of diagnosis in 2 patients, as shown in Table 6.

**TABLE 6:** Molecular characteristics of 33 patients with classic homocystinuria.

Patient	Genetic test	Variant coordinates	Amino acid change	Pathogenicity	Novelty	Zygosity	PMID	Reference
1	*CBS*	NM_000071.3: c.828+2‐828+ 3 delinsACACTTGCATCC	p.?	Likely pathogenic	Novel	Homo		
2	*CBS*	NM_000071.3: c.828+2‐828+ 3 delinsACACTTGCATCC	p.?	Likely pathogenic	Novel	Homo		
3	*CBS*	NM_000071.3: c.828+2‐828+ 3 delinsACACTTGCATCC	p.?	Likely pathogenic	Novel	Homo		
4	WES	NM_000071.3: c.1006C>T	p. (Arg336Cys)	Pathogenic	Known	Homo	20455263	16
5	WES	NM_000071.3: c.1006C>T	p. (Arg336Cys)	Pathogenic	Known	Homo	20455263	16
6	*CBS*	NM_000071.3: c.1006C>T	p. (Arg336Cys)	Pathogenic	Known	Homo	20455263	16
7	WES	NM_000071.3: c.1093C>T	p. (Gln365*)	Pathogenic	Known	Homo	32000841	17
8	*CBS*	NM_000071.3: c.1093C>T	p. (Gln365*)	Pathogenic	Known	Homo	32000841	17
9	WES	NM_000071.3: c.969G>A	p. (Trp323*)	Pathogenic	Known	Homo	21517828	18
10	Carrier testing	NM_000071.3: c.969G>A	p. (Trp323*)	Pathogenic	Known	Homo	21517828	18
11	Carrier testing	NM_000071.3: c.969G>A	p. (Trp323*)	Pathogenic	Known	Homo	21517828	18
12	WES	NM_000071.3: c.969G>A	p. (Trp323*)	Pathogenic	Known	Homo	21517828	18
13	*CBS*	NM_000071.3: c.(1566del)	p. (Lys523fs*)	Pathogenic	Known	Homo	11553052	19
14	*CBS*	NM_000071.3: c.(1566del)	p. (Lys523fs*)	Pathogenic	Known	Homo	11553052	19
15	*CBS*	NM_000071.3: c.(654G>A)	p. (Trp218*)	Pathogenic	Known	Homo	21517828	18
16	WES	NM_000071.3: c.969G>A	p. (Trp323*)	Pathogenic	Known	Homo	21517828	18
17	WES	NM_000071.3: c.969G>A	p. (Trp323*)	Pathogenic	Known	Homo	21517828	18
18	WES	NM_000071.3: c.969G>A	p. (Trp323*)	Pathogenic	Known	Homo	21517828	18
19	WES	NM_000071.3: c.969G>A	p. (Trp323*)	Pathogenic	Known	Homo	21517828	18
20	WES	NM_000071.3: c.969G>A	p. (Trp323*)	Pathogenic	Known	Homo	21517828	18
21	*CBS*	NM_000071.3: c.969G>A	p. (Trp323*)	Pathogenic	Known	Homo	21517828	18
22	*CBS*	NM_000071.3: c.969G>A	p. (Trp323*)	Pathogenic	Known	Homo	21517828	18
23	WES	NM_000071.3: c.828+1G>		Pathogenic	Known	Homo	10338090	18
24	*CBS*	NM_000071.3: c.969G>A	p. (Trp323*)	Pathogenic	Known	Homo	21517828	18
25	WES	NM_000071.3: c.969G>A	p. (Trp323*)	Pathogenic	Known	Homo	21517828	18
26	*CBS*	NM_000071.3: c.969G>A	p. (Trp323*)	Pathogenic	Known	Homo	21517828	18
27	WES	NM_000071.3: c.969G>A	p. (Trp323*)	Pathogenic	Known	Homo	21517828	18
28	WES	NM_000071.3: c.969G>A	p. (Trp323*)	Pathogenic	Known	Homo	21517828	18
29	WES	NM_000071.3: c.969G>A	p. (Trp323*)	Pathogenic	Known	Homo	21517828	18
30	WES	NM_000071.3: c.1006C>T	p. (Arg336Cys)	Pathogenic	Known	Homo	20455263	16
31	WES	NM_000071.3: c.654G>A	p. (Trp218*)	Pathogenic	Known	Homo	21517828	18
32	WES	NM_000071.3: c.654G>A	p. (Trp218*)	Pathogenic	Known	Homo	21517828	18
33	WES	NM_000071.3: c.654G>A	p. (Trp218*)	Pathogenic	Known	Homo	21517828	18

Abbreviations: CBS, cystathionine beta‐synthase; homo, homozygous; WES, whole exome sequencing.

**FIGURE 2:**
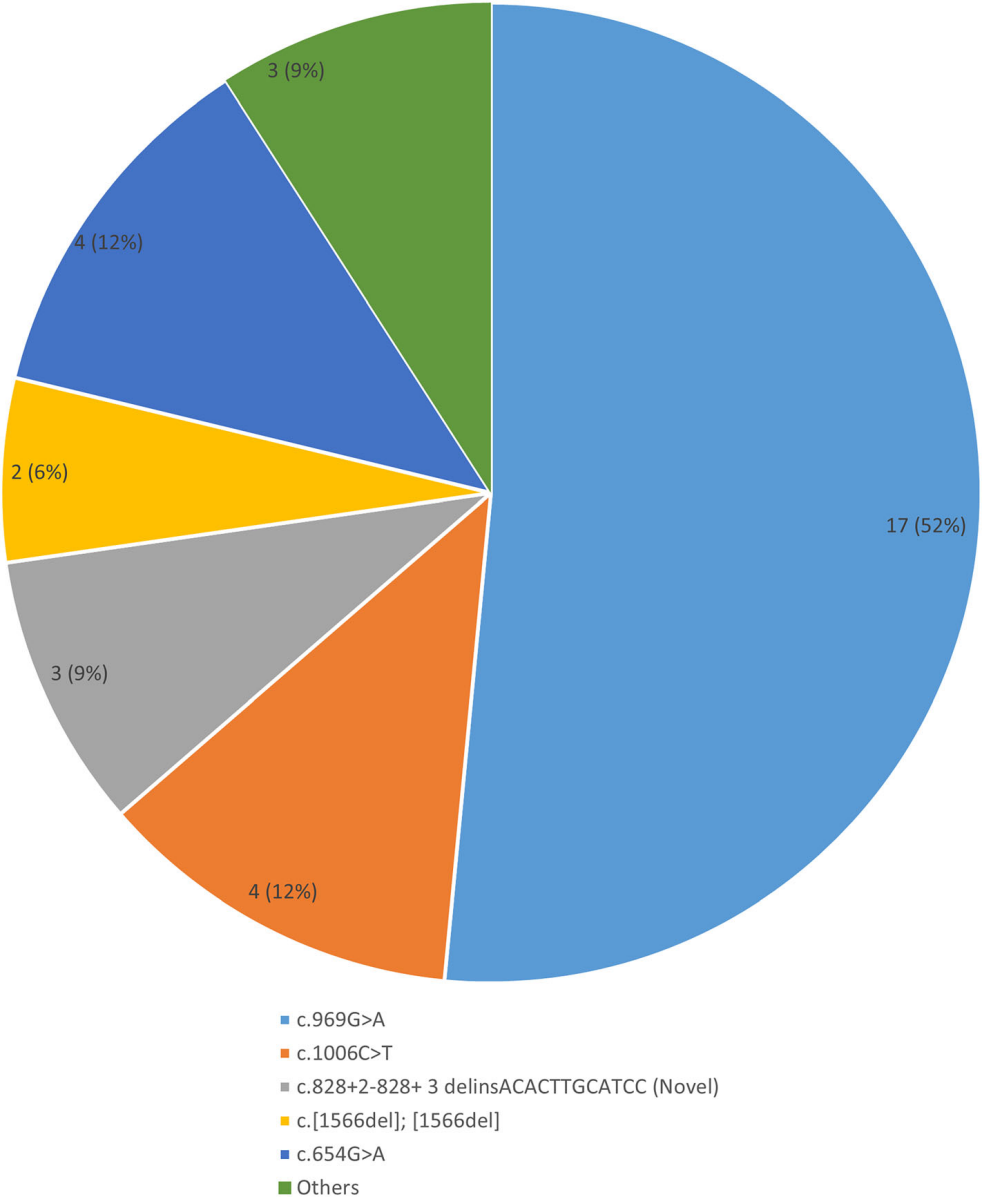
Type of variants in 33 patients with classic homocystinuria.

All identified variants in this cohort were homozygous and included missense, nonsense, deletion, duplication, insertion and splice site donor as shown in Figure 2 and Table 6. One novel variant (c.828+2‐828+ 3 delinsACACTTGCATCC) was observed in 3/33 patients (9%). A founder nonsense mutation, c.969G>A, p. (Trp323*) was detected in 17 patients (52%), all from one tribe.

#### 3.3.3 Imaging studies

The imaging studies included echocardiography, brain scans and carotid doppler scans.

##### Echocardiography

Thirteen patients were evaluated by an echocardiogram, nine of whom (69%) elicited abnormal findings, which were asymptomatic and generally mild. Abnormal findings comprised valvular abnormalities and ventricular remodeling. Eight of the nine patients had valvular regurgitation, with more than one valve most often involved. Among the eight patients, there were 15 valvular regurgitations found, five of them were mitral, and one was mitral valve prolapse. Furthermore, four patients (27%) had tricuspid regurgitation, four (27%) had aortic regurgitation, and a further two (14%) had pulmonary regurgitation.

In addition, two patients had ventricular remodeling, one of which was left ventricular hypertrophy, and the other was right ventricular dilation, which manifested in conjunction with tricuspid regurgitation and pulmonary regurgitation.

##### Brain imaging

Eleven patients had brain scans, including CT and MRI with or without venography, done on suspicion of cerebrovascular pathology. Eight patients had no abnormal findings. Two patients had cerebral venous thrombosis. One patient with poorly controlled HCU, intellectual disability and history of CVT presented with headaches, and vomiting. Her MRI brain showed diffusion restriction within the subcortical white matter and at lesser degree within the central tegmental tract representing likely myelin edema. Unfortunately, her methionine level was not measured during this episode.

##### Carotid doppler imaging

Three patients underwent carotid doppler scanning, one had carotid artery stenosis while the other two showed no abnormality.

### 3.4 Treatment

Treatment was fairly uniform among patients and generally followed the management and dosage guidelines. It included folic acid 5 mg per daily, hydroxocobalamin 1000 μg intramuscular weekly, pyridoxine 50 mg per day, and betaine 150 mg/kg/day or if necessary, to maximum of 3 g two times a day for adults.^8^ Furthermore, nutritional management and calculations was supervised by certified metabolic dieticians and included a low‐methionine special formula. Although all patients were treated with high dose of pyridoxine for more than a year and labelled as pyridoxine nonresponsive HCU, they all continued on pyridoxine throughout their treatment course. Furthermore, all patients were given a low‐methionine special formula (hominex) with variable compliance. Generally, compliance with nutritional and medical treatment was inconsistent across the patients.

## 4 DISCUSSION

The phenotype of the individuals with classic homocystinuria in this cohort has shown a wide‐range clinical spectrum, as was expected, with some patients relatively unaffected, and many others profoundly affected by several complications. However, only five patients among our 33 were diagnosed through NBS, and it is hoped that morbidity and mortality can be significantly diminished in the future with the expansion of our newborn screening program to include homocystinuria in 2020, in keeping with the literature.^12^, ^20^

Although the five patients diagnosed with NBS had better outcomes than the rest of the study subjects, this finding is limited by the small number of newborn screened patients and the short duration of follow‐up thereafter (Table 2). Therefore, larger studies with longer follow up time will better address the outcome of HCU.

Similar to our study, Zaidi et al.^18^ reviewed 32 patients with classic homocystinuria attending a tertiary center in Saudi Arabia. The range of the age at diagnosis of their patients was 3 weeks to 20 years which was echoed by our data. However, none of their patients were diagnosed by universal NBS as this initiative had not been developed by then in Saudi Arabia.

Zaidi et al. presented data on complications and 12 patients had neuropsychiatric deficits, five of them had ADHD compared to our 23 neuropsychiatric deficits including seven ADHD diagnoses.^18^ Lens deficits are one of the most pertinent findings in HCU patients and manifested in 68% of the patients in the Zaidi et al. cohort compared to 70% of our patients.^18^ Despite the difference in age group between our cohort and Zaidi et al. study, the pattern of complication is more or less similar.^18^ This is probably because the two studies were conducted in the same highly consanguineous community in Saudi Arabia.

A similar, older Dutch study by Kluijtmans et al.^21^ presented the complications of a sample of 29 patients with homocystinuria at diagnosis. Since our study sample had a mean age of 7.5 year and their mean age of 26 year at diagnosis, their cohort had a more severe complications profile, suggesting a correlation between late diagnosis and a more extensive complication profile.^21^ In their study, 90% had a late diagnosis (through a complication rather than family screening/newborn screening) compared to our 85% non‐NBS diagnosis.^21^ The complications profile was particularly more extensive in ophthalmic and musculoskeletal complications, as 86% of their patients presented with visual defects/refractory errors compared to our 70% who suffered from lens dislocation/subluxation.^21^ Furthermore, osteoporosis and marfanoid features was a complication in 6% and 30% of our patients respectively, compared to the respective complication rates of 52% and 62% in their sample. In addition, four patients in their sample of 29 had psychosis, which was not a feature found in any of our patients.^21^ Interestingly, despite this, the rate of intellectual disability among their cohort was lower than ours which was 42%, and the proportion of patients with thromboembolic complications was comparable.^21^ One potential confounder between our study and the Dutch study is the gross genotypic dissimilarities between the two study populations, as the two samples bear not a single mutation in common.^21^

A recent Qatari study by Al‐Dewik et al.^22^ categorized 126 patients into those with NBS diagnosis, those with family screening diagnosis, and those with late (complicated) diagnosis. The patients in this sample had earlier diagnosis than ours, with 55% having late diagnoses compared to our 85%.^22^ Similar to the Dutch study, Qatari patients with late diagnosis had a substantially higher rate of ocular, marfanoid manifestations and intellectual disability, at 92%, 98.5% and 79% respectively.^21^, ^22^

Thromboembolic complications in the Qatari study interestingly only affected 4% of those patients, significantly lower than our study which showed that 21% of the study group had thromboembolic events.^22^ In comparison, a multicenter observational study on 158 patients conducted by Yap et al. showed 17 vascular events, which occurred in 12 (7.6%) of the patients.^23^ Moreover, unlike our study whose thromboembolic complications were limited to DVT and cerebral venous thrombosis, this multicenter study also had myocardial infarction and pulmonary embolism among the thromboembolic complications, and abdominal aortic aneurysm among the vascular complications.^23^ This difference between the two samples may be a reflection of the age differences, as this patient sample has a mean age of 42.5, ranging from 18 to 67.^23^ Thromboembolism is expected in patients with poor metabolic control irrespective of the age as hyperhomocystenemia is a known risk factor for coagulopathy.^3^ However, aortic aneurysm and myocardial infarction are unusual features of HCU.^23^

The molecular findings of our cohort revealed eight variants, one of which is novel. The variants c.969G>A and c.1006C>T comprised 70% of the mutations in our cohort compared to 86% of the variants in Zaidi et al. study.^18^ This genotypic similarity is not unexpected as the two cohort were demographically similar and both from highly consanguineous community. Furthermore, the founder variant, c.969G>A was reported in 17/33 (52%) of our patients compared to 71% in Zaidi et al. cohort.^18^ Interestingly, all these 17 patients were from a single tribe. Given this finding, it would be cost‐effective to request a targeted mutation study to confirm HCU in individuals presented with high homocysteine from this particular tribe rather than ordering sequencing of the whole CBS gene. Despite the genotypic similarities between the two samples, the clinical manifestations showed many differences, and this may also reflect a high level of variation in gene expression. This conclusion can further be substantiated by the genotype–phenotype variability in our cohort. Similarly, other cohorts have shown that the pattern of complications was pretty similar among patients carrying different mutations.^18^, ^20^, ^21^ Also, a systematic review has shown that even among geographically proximate demographics, genotypic differences in homocystinuria patients are vast with variable phenotypes.^24^ It seems that the rate limiting factors for reducing the development of complications is not the genotype but the early diagnosis together with the good metabolic control with Hcy levels within the acceptable range.^8^

We identified the homozygous novel deletion–insertion variant c.828+2‐828+ 3 delinsACACTTGCATCC in the CBS gene in three siblings. This variant results in a loss of the donor splice site of exon 9 and therefore a skip of exon 9 is very likely. Its minor allele frequency (MAF) is <0.0005.^25^ This variant has not been described before in the literature. However, a different change leading to the loss of the donor splice site of exon 9, c.828+1 G>A was described as pathogenic variant in the HGMD database.^25^

In our study, the failure to institute timely diagnosis was also an adversity, since most of our patients were diagnosed on clinical ground and mainly with complications and few presented before the onset of symptoms either as a result of NBS or after diagnosis of an index case in the family. The implementation of a NBS program, as well as educating families and physicians on screening for patients with a family history, can lead to early diagnosis, which ultimately improves the biochemical control and outcomes.^22^ In particular, the prevalence of intellectual disability among our patients was a tragedy, however, with the implementation of our NBS program we are hopeful for an improvement, as NBS‐diagnosed patients have better intellectual outcomes as reflected by our data and other cohort.^22^, ^26^

The biochemical profile of our patients showed that both total Hcy and methionine were consistently elevated at the time of the initial diagnosis and during the treatment course for patient diagnosed on NBS and those diagnosed on clinical grounds. The high total Hcy and methionine at the time of the diagnosis is expected for patients diagnosed on clinical grounds as they are likely to be on regular protein containing diet before the diagnosis. Also, the elevated total Hcy and methionine levels at the time of diagnosis of newborn screened infants can be explained by the fact that the biochemical confirmation which comprise initial and recall methionine tests in addition to the total Hcy confirmation takes few weeks in our setting and during this time the infants are usually on unrestricted protein formula. The mean total Hcy level during follow up, which mirrors the metabolic control of HCU, was significantly lower in patients diagnosed by NBS (107 ± 45) μmol/L compare to those who were not diagnosed by NBS (240 ± 149) μmol/L, *p* < 0.045. This shows that the patients diagnosed by NBS are likely to be compliant with medical and nutritional management especially the intake of low protein diet and the betaine. This is unlike patients who were not diagnosed on NBS who tend to be older and already used to regular protein containing diet. Shifting these patients to low protein diet is challenging especially during adolescence and also because of the taste of the special formula which is not liked by many patients.^8^

We observed that unlike total Hcy, methionine level during follow up was more or less similar in patients diagnosed by NBS and those who were not. This can be explained by the fact that methionine is not a good marker for follow up of HCU and does not indicate accurately the metabolic control of patients.^8^ This is because patients not compliant with protein restriction and not taking betaine are expected to have high methionine. Also, patients on special formula with low protein and betaine may have high methionine as betaine converts homocysteine to methionine by the enzyme betaine‐homocysteine S‐methyltransferase and therefore increases the level of serum methionine.^3^, ^8^

The echocardiographic evaluation of our patients revealed features of asymptomatic abnormalities, with mild valvulopathy being particularly common among them (which led to the misdiagnosis of Marfan syndrome in two of our patients), with mitral and tricuspid regurgitation being the most commonly involved. This mirrors a study carried out by Kalil et al. on 14 patients with HCU, whereby six patients had abnormalities, with three having tricuspid regurgitation and two having mitral regurgitation and mitral prolapse.^27^ Another similarity is the presence of remodeling. One patient in that study had atrial remodeling whereas two of ours had ventricular remodeling.^27^ Nevertheless, major valvular defects and ventricular hypertrophy or dilatation are not cardinal features of HCU and their pathophysiology is not clearly linked to hyperhomocystinemia and poor metabolic control.^8^, ^27^ Mostly the cardiac findings of our patients are likely to be coincidental.

While little is known about the pathophysiology of complications associated with HCU apart from thromboembolism, experimental data from patients and animal models indicate that oxidative stress may play a role in the pathogenesis of these complications.^28^ In this case, studies have demonstrated that people with HCU have lower antioxidant defense.^28^ Additionally, in animal models as well as in patients with HCU, there have been observations of elevated indicators of oxidative damage to protein, lipids and DNA in the blood, skeletal muscles, liver and brain, most likely secondary to increased production of free radicals.^28^ This accumulating metabolite may be the cause of the oxidative damage seen in both human and animal model. This disturbance of redox balance may have a role in the tissue injury seen in HCU.^28^ Interestingly, our study demonstrated a sustained irreversible complication in patients diagnosed on clinical ground, mostly late while none of the early diagnosed ones by NBS have had a complication. This observation supports the fact that the early diagnosis and initiation of the therapy may halt the free radicals formation, oxidative stress, cellular damages and preserve the tissues from longstanding injuries.

One of the limitations of our study is that patients have not undergone formal pyridoxine responsiveness tests using standard protocols.^8^ However, the treating physicians classified all patients as pyridoxine unresponsive based on failure to lower total Hcy level despite high dose of pyridoxine used for all patients in the first year after diagnosis. Moreover, the pattern of mutations reported from the Middle East, which are virtually similar to our patient's genotype, are largely unresponsive to pyridoxine.^18^, ^22^, ^24^, ^25^ Based on the assumption of the treating physicians of our patients in addition to the genotype we can conclude that patients recruited in this study are pyridoxine unresponsive. Nevertheless, pyridoxine responsiveness is a very important independent variable since it is a determinant of clinical outcome and severity and all patients with HCU should strictly undergo formal test to determine their responsiveness to pyridoxine beyond doubt.^8^

Other limitation of this study is the relatively small number of patients studied which makes it difficult to draw a firm statistical correlation across variables. Additionally, it makes it difficult to account for random error, especially given the wide‐range findings across the patients and the retrospective nature of the study. Also, the neurodevelopmental evaluation of our patients was largely based on the judgment of the treating physician and formal IQ assessment was done in four patients only which adds to the limitation of this study.

In conclusion, we described the clinical, biochemical and molecular characteristics and the clinical course of all patients with HCU evaluated in our center. Compared to the patients diagnosed on clinical ground, we have shown that patients diagnosed on NBS are less likely to suffer from complications such as ophthalmic, neuropsychiatric, skeletal and thromboembolism.

## AUTHOR CONTRIBUTIONS

All authors contributed to the literature review, data collection, data analysis and drafting and reviewing the manuscript. The contributions of Dr. Arwa Ali Hudaisy and Dr. Norah Sarhan in data collection is also acknowledged.

## CONFLICT OF INTEREST STATEMENT

The authors declare no conflicts of interest.

## ETHICS STATEMENT

All human research conducted was in accordance with the required ethical standards and was approved by the Institutional Review Board of Price Sultan Military Medical City, Riyadh, Saudi Arabia, Ethical approval reference.^27^

## Data Availability

All this research project data is available and ready to share on demand.
